# Work Flexibility and Work-Related Well-Being

**DOI:** 10.3390/ijerph18063254

**Published:** 2021-03-21

**Authors:** Tapas K. Ray, Regina Pana-Cryan

**Affiliations:** Economic Research and Support Office (ERSO), National Institute for Occupational Safety and Health (NIOSH), Centers for Disease Control and Prevention (CDC), 1150 Tusculum Avenue, C-24, Cincinnati, OH 45226, USA; rfp2@cdc.gov

**Keywords:** future of work, healthy work design and well-being, work organization, work arrangements, demographic shifts, compensation and benefits, occupational stress

## Abstract

Work organization practices, including work flexibility, are changing and can affect worker well-being. Common work flexibility types include working at home, taking time off when needed, and changing one’s work schedule. Given the changes in and the importance of work flexibility, the study assesses its prevalence and association with worker well-being in the United States. We used 2002–2018 General Social Survey—Quality of Worklife (GSS-QWL) data, descriptive statistics, and regression analyses to assess the reported likelihood of job stress, job satisfaction, healthy days, and days with activity limitations among workers reporting work flexibility. The prevalence of work flexibility remained relatively stable during the period examined. Working at home increased the likelihood of job stress by 22% and job satisfaction by 65%. Taking time off decreased the likelihood of job stress by 56% and days with activity limitations by 24%, and more than doubled the likelihood of job satisfaction. Changing one’s schedule decreased the likelihood of job stress by 20% and increased the likelihood of job satisfaction by 62%. This study used all the available data from GSS-QWL and demonstrated the ongoing importance of work flexibility for well-being.

## 1. Introduction

Work organization practices, including non-standard work arrangements, flexible schedules, and the use of technology and automation, are going through substantial changes globally [1,2,3,4]. In addition, the demographic, technological, and economic changes that have been molding the ways work is organized seem more pronounced than in the immediate past [5,6]. In recent years, the US working population has aged, and gender gaps in labor force participation are slowly being bridged, with women joining the workforce in large numbers [5,7]. During the past year, the gap in women’s labor force participation increased, but it is not clear yet if the gap will persist [8]. These trends, combined with technological advancements that accommodate rapid reductions in certain types of economic transaction costs such as labor productivity monitoring costs, have altered organizational practices, including work flexibility [2,9].

While there is no standard definition of work flexibility [10], it often includes characteristics in terms of how, where, when, and for how long work is done [10,11,12,13], and it has been a priority need for some workers and employers alike [14]. The three most common types of flexibility available to US workers are the ability to: change work location; take time off; and change their work hours [10,11,12,13,14,15].

Work flexibility can have positive and negative consequences for workers and their families, employers, and society overall [15,16,17,18,19,20,21,22,23,24,25,26,27,28]. Work flexibility accommodates workers by enabling them to allocate resources between work and non-work domains according to their preferences [9,10,11,12,13,14,15]. For example, workers with access to flexible work schedules can ease some of the chronic time pressures and conflicts imposed by their non-work responsibilities. Workers seek flexibility to address their personal and family needs, including childcare, eldercare, schooling, and healthcare [14]. The available evidence supports that work flexibility in terms of location [17] and work hours [19] gives workers some sense of job control, improves their engagement, and increases their job satisfaction, thereby improving their health and well-being [17,18,19]. Bond and Galinsky [2011] found that both high- and low-wage workers valued work flexibility in terms of work schedule, and that flexibility was a statistically significant contributor to job engagement and job satisfaction [23]. Golden (2008) found that flexibility was positively related to happiness [24]. Using US data from the General Social Survey (GSS), that study found that the contribution of flexibility in terms of control over schedule on happiness was higher for wage workers compared to salaried workers. Kim et al. (2020) used 2002–2014 GSS data to examine the associations between flexibility, measured with flexible schedules and working at home, and worker well-being, measured with job satisfaction, job stress, daily fatigue, and work-to-family conflict. The authors found benefits of flexible schedules and some benefits of working at home, even though working at home to catch up on work had disadvantages for worker well-being. The authors concluded that there were potential advantages and unintended consequences of different flexibility arrangements for workers, and that these implications might differ by gender [25]. Additional available evidence supports that some of the work–family conflicts associated with contingent work can be outweighed by the benefits of work schedule flexibility [16], but also that the blurring of work and non-work life boundaries can have negative consequences for workers and their families [20].

Employers also benefit from more productive and engaged workers [19,21,22], and work flexibility increases engagement, as mentioned above. From society’s perspective, flexibility, especially location flexibility, can accommodate short- or long-term operational continuity if workplace closures become necessary.

Despite the importance of work flexibility for both workers and employers, the understanding of its prevalence over the years remains limited. A 2016 survey of 920 employers with 50 or more employees [26] found that, between 2012 and 2016, only 4 of 18 types of flexibility examined statistically significantly changed. These four types of flexibility, expressed as the percentage of employers allowing at least some employees access to them, included: returning to work gradually after childbirth or adoption (increased from 73% in 2012 to 81% in 2016); receiving special consideration after a career break for personal or family responsibilities (increased from 21% in 2012 to 28% in 2016); working some of their regular paid hours at home on a regular basis (increased from 33% in 2012 to 40% in 2016) and taking time off during the workday to attend to important family or personal needs without loss of pay (decreased from 87% in 2012 to 81% in 2016). The change in the percentage of employers allowing at least some employees to change starting and finishing times on a daily basis was not statistically significant during the same time period (increased from 39% in 2012 to 42% in 2016). Data from the Bureau of Labor Statistics (BLS) American Time Use Survey (ATUS) show an upward trend in flexibility in terms of access to working at home and access to flexibility in schedules. In 2003 and 2007, 18.6% and 19.9% worked at home, respectively [29]. According to the 2017–2018 ATUS, 28.8% of workers could work at home and 24.8% did work at home [30]. Note, however, that survey questions have changed over time and pre- and post-2017 data are not fully comparable [31]. Fifty-six percent of workers were able to adjust their work schedules or location of their main jobs instead of taking time off from work in 2011 [32]. During 2017–2018, 57% of wage and salary workers had a flexible schedule, in which they were able to vary the times they began and stopped working [33]. In 2020, paid sick leave was available to 78% of all workers [34], an improvement compared to 2011(55%) [35]. It is evident from the information presented above that there is a growing interest in studying work flexibility, particularly in terms of its contribution towards overall worker well-being.

The purpose of this study is to understand flexibility trends in the US and its association with well-being. This study examined the following research questions: (1) What are the trends of flexibility over time (using descriptive analysis)? (2) What are the population prevalence rates by flexibility indicators and selected demographic and socioeconomic characteristics (using descriptive analyses)? (3) What are the well-being outcomes of flexibility (using logistic and zero-inflated negative binomial regressions, and controlling for covariates)? The study used US data from GSS—Quality of Worklife (QWL) to assess the prevalence of work flexibility and its association with worker well-being. This research advances the understanding of work flexibility and its effects in several ways, including by expanding on the findings by Kim et al. (2020). We used more years of GSS-QWL data than previous studies. We included three indicators of flexibility: (i) ability to work at home (location flexibility), (ii) ability to take time off for family needs (leave flexibility), and (iii) ability to change starting and finishing times (schedule flexibility). We used four indicators of well-being, including (i) job stress, (ii) job satisfaction, (iii) number of healthy days during the previous thirty days, and (iv) number of days with activity limitations due to poor health in the last thirty days. In addition, this study used descriptive statistics on flexibility by year, demographic and socioeconomic characteristics, industry, work arrangement, and four indicators of well-being.

The sections that follow present materials and methods, results, discussion including limitations, and conclusion.

## 2. Materials and Methods

### 2.1. Data

As mentioned earlier, this study used GSS-QWL data (for details, see NIOSH 2013 [36]). Funded by the National Science Foundation, GSS is a biannual, nationally representative cross-sectional survey of US households conducted through face-to-face personal interviews by the National Opinion Research Center (NORC). GSS utilizes a multistage probability design yielding a representative sample of the civilian, non-institutionalized, English-speaking, US adult population [37]. Developed by the National Institute for Occupational Safety and Health (NIOSH) with contributions by its partners, the QWL module assesses an array of psychosocial working conditions, such as job demands and autonomy [37], and quality of worklife topics among GSS respondents who are either employed or looking for work at the time of the survey. The QWL module is administered every four years.

This study used GSS-QWL data for all currently available years, including 2002, 2006, 2010, 2014, and 2018. We used weights provided by GSS-QWL, so that the data represent the US working population. This is important because we assess the prevalence of flexibility practices across the years. For the regression analysis, we used pooled data from all five years.

We used GSS-QWL responses from only those who reported being employed at the time of the survey. After adjusting for sampling probabilities, including subsampling for non-respondents (approximately 70% response rate each survey year) and correcting for the number of adults in the household, the nationally representative sample we used in our analyses consisted of approximately 7400 observations distributed more or less evenly across the five years.

#### 2.1.1. Indicators of Flexibility

We assessed work flexibility using location, leave, and schedule flexibility variables. We measured location flexibility by the frequency of working at home according to responses to the question: *How often do you work at home as part of your job?* Due to the analytical approach we followed, we converted the 6-point Likert scale responses, as well as all other Likert scale responses to questions we used in this study, into a dichotomous variable, following Grosch et al. (2006) and Ray et al. (2017): “work at home” for responses always, more than once a week, and about once a week, and “do not work at home” for responses once a month, a few times a year, and never [37,38]. We measured leave flexibility according to responses to the question: *How hard is it to take time off during your work to take care of personal or family matters?* We converted the 4-point Likert scale responses into a dichotomous variable: “take time off” for responses not at all hard and not too hard, and “do not take time off” for responses somewhat hard and very hard. We assessed schedule flexibility according to responses to the question: *How often are you allowed to change your starting and quitting times on a daily basis?* We converted the 4-point Likert scale responses into a dichotomous variable: “change schedule” for responses often and sometimes, and “do not change schedule” for responses rarely and never. This question was not included in the 2018 QWL module, resulting in fewer available observations.

#### 2.1.2. Indicators of Well-Being

We measured job stress using responses to the question: *How stressful is your work?* We converted the 5-point Likert scale responses into: “stressed” for responses always and often, and “not stressed” for responses sometimes, rarely, and never. We measured job satisfaction according to responses to the question: *All in all, how satisfied would you say you are with your job?* We converted the 4-point Likert scale responses into: “satisfied” if very satisfied and somewhat satisfied, and “not satisfied” if not too satisfied and not at all satisfied.

We also used three items from GSS-QWL that assess the Centers for Disease Control and Prevention (CDC) Health-related Quality of Life (HRQOL)-4 index. Developed in the 1980s, the HRQOL-4 has been used to derive metrics for government-wide initiatives such as Healthy People 2010 and 2020 [39] and assess the health status of the US population both at the national and state levels. Variables from the HRQOL-4 have been used in national-level surveys such as CDC’s Behavioral Risk Factor Surveillance System and the National Health and Nutrition Examination Survey. The four core questions from the HRQOL-4 were: (1) *Would you say that in general, your health is excellent, very good, good, fair, or poor?*; (2) *Now thinking about your physical health, which includes physical illness and injury, for how many days during the past 30 days was your physical health not good?*; (3) *Now thinking about your mental health, which includes stress, depression, and problems with emotions, for how many days during the past 30 days was your mental health not good?*; and (4) *During the past 30 days, for about how many days did poor physical or mental health keep you from doing your usual activities, such as self-care, work, or recreation?* Out of the responses to these four questions, we used responses to the first one as a covariate to control for the overall health status of the respondent. We converted Likert scale responses to dichotomous: “health status good” for excellent, very good, and good, and “health status not good” for fair and poor health status. To measure healthy days, one of our well-being outcome variables, we used responses to questions 2 and 3. We added the number of days reported in responses to these questions to estimate the number of days within the previous 30 days for which a respondent was not in good physical and mental health. If the sum exceeded 30 days, we recorded 30 days. The sum of these “unhealthy days” when subtracted from the overall last 30 days, produces the reported number of “healthy days” by a respondent during this period. This construct of healthy days, although simple, has been tested for construct validity, concurrent validity, and HRQL predictive validity [40]. We used responses to question 4 to estimate “days with activity limitations”.

#### 2.1.3. Other Indicators of Interest

This study assessed flexibility practices by industry by grouping respondents into ten broad industries based on broadly defined National Occupational Research Agenda (NORA) industrial sectors (for more information, see CDC 2018B [41]).

In order to assess flexibility practices across different work arrangements, we distributed the study sample into five mutually exclusive groups based on responses to the question: *How would you describe your employment arrangement in your main job?* Response categories were: (1) independent contractor/independent consultant/freelance worker (contractor), (2) on-call worker/works only when called (on-call), (3) paid by temporary agency (temporary), (4) working for a contractor who provides workers and services to others under contract (under contract), and (5) regular permanent employee (standard). We estimated the number and proportion of workers in each arrangement category according to their responses to the flexibility questions, as described above.

The available evidence supports that family–work interference is associated with job stress [42]. To control for spilling over of “outside work” stress on stress at work, we controlled for responses to the family–work interference question: *How often do the demands of your family interfere with your work on the job?* We converted the 4-point Likert scale responses into a dichotomous variable: “Family interferes with work” for responses frequently and sometimes, and “family does not interfere with work” for responses rarely and never.

In the GSS-QWL, respondents are asked whether they consider their work part-time or full-time. Approximately 17% of our sample responded working part-time. We found that, compared with full-time workers, part-time workers had higher flexibility in terms of being able to take time off and change their schedules, but had lower flexibility in terms of working at home. The difference was statistically significant for the ability to take time off. In terms of the well-being variables we examined, we found that part-time workers reported being less stressed. In this study, our objective was to assess whether flexibility was associated with well-being. We assumed that the effect of part- and full-time work was endogenous through the flexibility itself in measuring the effect on job stress. To check our assumption, we included working part-time as a covariate in our regression analyses of the effect of flexibility variables on job stress and found no statistically significant changes in our estimated odds ratios. Hence, we did not include the variable in our current analysis. However, we controlled for hours worked per week. The GSS asks respondents about their usual number of work hours in a week. We used that as a control variable.

### 2.2. Analyses

#### 2.2.1. Descriptive Analyses

We conducted descriptive analyses to assess: (1) trends of flexibility over time; (2) population prevalence rates by flexibility indicators and selected demographic and socioeconomic characteristics (age, sex, race and ethnicity, education, family income, health status, marital status, family-work interference, and hours worked per week); (3) well-being outcomes (job stress, job satisfaction, healthy days, and days with activity limitations); (4) flexibility by NORA sector; and (5) flexibility by work arrangement.

#### 2.2.2. Regression Analyses

Next, we assessed the impact of individual work flexibility variables on work-related well-being variables using several regression models and controlling for age, sex, race and ethnicity, education, family income, health status, marital status, family–work interference, and hours worked per week. We used two types of regression analyses depending on the nature of the dependent variable: for categorical outcome variables such as job stress and job satisfaction, we used logistic models; for the healthy days and days with activity limitations, which are count variables, we used zero-inflated negative binomial models as described below. Age and the logarithmic transformation of family income were continuous variables, education was a categorical variable (1–7 years of school, 8 years of school, 9–11 years of school, high school graduate, 13–15 years of school, bachelor’s degree, postgraduate), and the rest of the variables were dichotomous. The reference group for sex was “male”, for race and ethnicity “non-Hispanic White”, for health status “not good”, for marital status “married and living with spouse”, and for family–work interference “family does not interfere with work”.

Logistic regressions: We estimated the effect of flexibility variables on job stress and job satisfaction, controlling for the covariates mentioned above. Previous studies showed that the demographic variables we included might contribute to stress and affect physical and mental health [23,24,25]. Previous studies also showed the importance of work–family conflict for stress [27,28]. As the objective of this study was to assess how work flexibility was associated with job stress and satisfaction, we did not control for specific psychosocial working conditions separately in the regression analysis. We acknowledge that there will be direct and indirect effects on job stress and satisfaction through stressful psychosocial working conditions such as job demand and control.

Zero-inflated negative binomial regressions: Following Zhou et al. (2014), we used zero-inflated negative binomial two-part models to estimate the effect of flexibility on healthy days and activity limitations, controlling for the covariates mentioned above [43]. As many study participants may have a zero count of healthy days or days with activity limitations, the data are not normally distributed, and the positive skew in their distribution cannot be resolved by data transformation. The zero-inflated negative binomial model provides a way of modeling the excess number of zeros as well as allowing for count data that are skewed and over-dispersed. It is a two-part model that combines the logistic regression model and the negative binomial model. The first part, logistic regression for excess zeros, predicts the probability of having excess zero unhealthy days or days with activity limitations. The second part, negative binomial regression for the full range of counts, including random zeros, predicts the frequency of the unhealthy day or day with activity limitation count. Incidence rate ratios (IRRs) derived from the coefficients of the negative binomial random effects regression model can be interpreted in a way similar to interpreting odds ratios.

## 3. Results

### 3.1. Descriptive Analyses

**Figure 1:**
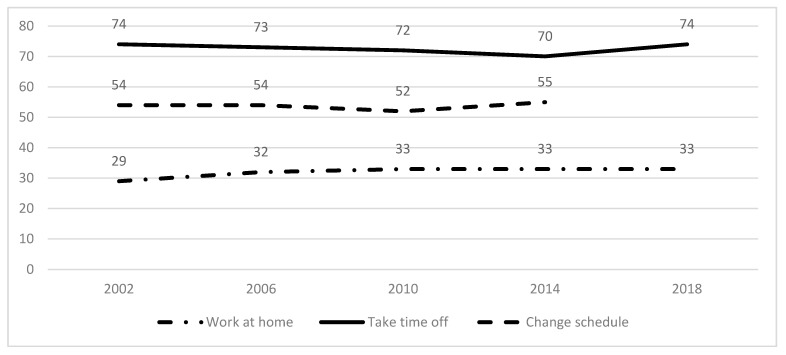
Prevalence of work flexibility over time (%), GSS-QWL data, 2002–2018.

Overall, the prevalence of work flexibility changed minimally during 2002–2018. The ability to work at home increased from 2002 (29%) to 2018 (33%) and the percentage of workers working at home remained almost the same from 2010 onwards. In comparison, the ability to take time off increased from 2010 (72%) to 2018 (74%), and the ability to change one’s schedule increased from 2010 (52%) to 2014 (55%). This is shown in Figure 1.

Table 1 presents the prevalence of work flexibility by demographic and socioeconomic characteristics within the study population and several work flexibility indicators. Women accounted for 51.7% of the respondents. Among those who worked at home, 48.9% were women. In addition, 50.8%, and 48.8%, respectively, of those who reported being able to take time off and change their schedule were women. Hispanics accounted for 10.6% of all respondents, and 7.4%, 9.7%, and 8.4%, respectively, of the respondents reporting being able to work at home, take time off, and change their schedule. Among the respondents who answered the question on race and ethnicity, 66.0% were non-Hispanic Whites, and among those with location, time, and schedule flexibility, 75.9%, 67.5%, and 74.0%, respectively, were non-Hispanic Whites. The percentage of those reporting that they were able to work at home increased with increasing levels of education, starting after 8 years of school. The pattern was less straightforward for the ability to take time off and change one’s schedule by level of education, with the percentages increasing after 8 years of school and decreasing after 15 years of school. Similar patterns were observed for family income categories, with flexibility increasing with income up to USD 50,000, then decreasing, and finally increasing again for income above USD 75,000. Those who were married and living with their spouse accounted for 47.3% of those who responded to the marital status question but reported more access to location, time, and schedule flexibility than those who were not married and living with their spouse (56%, 48.4%, and 51.4%, respectively). Of those who responded to the family interference question, 28.7% indicated that family interfered with work, and 66.1% of those who worked at home, 43.0% of those who took time off, and 60.0% of those who changed their schedule, respectively, reported that family interfered with work. Finally, of those who responded to the hours worked per week question, those who reported working at home worked a mean of 43.5 h and those who took time off a mean of 40.4 h.

Table 2 presents descriptive results for well-being by flexibility indicator. While 31.1% of respondents to the job stress question reported being stressed, 36.0% of those who responded working at home reported job stress. Of those who responded to the job satisfaction question, 89.8% reported being satisfied and at least 92.3% of those who had any type of flexibility reported being satisfied. Overall, more respondents had the ability to take time off, followed by those who could change their schedule, then those who had the ability to work at home. In terms of healthy days and days with activity limitations, there were small differences across flexibility types.

Table 3 presents results on flexibility by NORA sector. There was no single sector that dominated over the others across all the types of flexibility. Even though results by sector were not significant in terms of flexibility, we examined them to address NIOSH research priorities. Within sectors, the highest percentage reporting working at home were in agriculture, forestry, and fishing, followed by services; taking time off in construction followed by services; and changing their schedule in construction and agriculture, forestry, and fishing. Sectors in which workers reported having the least ability to work at home included mining (10.5%) and oil and gas extraction (11.1%), in which workers also reported having the lowest ability to change their schedule (16.7%). Across sectors, services accounted for 46.4% of those who responded to the question on sectors.

Table 4 presents results on flexibility by work arrangement. Compared to those in other work arrangements, contractors reported the highest percentages for all flexibility indicators. A higher percentage of on-call workers reported being able to change their schedule, but a lower percentage of those workers reported being able to work at home than those in standard arrangements. Those working for temporary help agencies reported the lowest percentages of ability to work at home (0.2%), take time off (65.8%), and change their schedule (33.9%).

### 3.2. Regression Analyses

Table 5, Table 6 and Table 7 report results on the associations between flexibility and well-being indicators. The first two columns of each table include findings for reported job stress and job satisfaction expressed as odds ratios. The next two columns of results include findings for reported healthy days and days with activity limitations expressed as incidence rate ratios (IRRs).

Table 5 presents the results for the ability to work at home. Working at home was associated with a 22% increase in job stress and a 65% increase in job satisfaction. Women were 38% more likely to report job stress and 5% less likely to report healthy days than men. Hispanic workers were 32% less likely to report job stress, 5% more likely to report healthy days, and 39% less likely to report days with activity limitations than non-Hispanic Whites. Black workers were 40% less likely to report job stress and 4% more likely to report healthy days, and Asian workers were 36% less likely to report less job stress and 4% more likely to report healthy days than non-Hispanic Whites. The likelihood of reported job satisfaction increased with family income, while the likelihood of reported healthy days increased and the likelihood of reported days with activity limitations decreased. The odds of job stress decreased by 36%, and the odds of job satisfaction increased more than two times as health status increased. Higher health status was associated with a 16% increase in healthy days and a 50% decrease in days with activity limitations. Being married and living with a spouse was associated with a 5% increase in job stress, a 5% decrease in job satisfaction, and a 1% decreased in the likelihood of reporting healthy days. For those reporting that family interfered with work, the likelihood of reporting job stress increased by 35%, job satisfaction decreased by 17%, heathy days decreased by 2%, and days with activity limitations increased by 9%. More hours worked were associated with a 3% increase in job stress, and 1% increase of job satisfaction, and an 11% increase in days with activity limitations.

Table 6 shows the results for the ability to take time off. Taking time off decreased the reported likelihood of job stress by 56%, more than doubled the reported likelihood of job satisfaction, increased the reported likelihood of healthy days by 2%, and decreased the reported likelihood of days with activity limitations by 24%. Women were 31% more likely to report job stress than men, 4% less likely to report healthy days, and 9% more likely to report days with activity limitations than men. Hispanics and non-Hispanic Blacks were less likely to report job stress and more likely to report healthy days compared with non-Hispanic Whites. Increases in education level and family income were associated with an increased likelihood to report job stress, job satisfaction, and healthy days. Hours worked per week did not result in substantial changes in work flexibility.

We report results on the association between the ability to change one’s schedule and well-being in Table 7. The ability to change schedule decreased the odds of job stress by 20% and increased the odds of job satisfaction by 62%. Compared to men, the likelihood of women to report job stress increased by 32% and the likelihood of women to report healthy days decreased by 5%. Compared to non-Hispanic Whites, Hispanic and non-Hispanic Black workers who could change their schedule were less likely to report job stress and more likely to report healthy days. Those who could change their schedule reported increases in job stress with increases in education and income and decreases in job stress with improvements in health status. Compared to those who reported their health status was not good, those who reported their health status was good were more than twice as likely to report job satisfaction, more likely to report healthy days, and less likely to report days with activity limitations. Those with family–work interference were more likely to report job stress, healthy days, and days with activity limitations and less likely to report job satisfaction. Hours worked per week did not result in substantial changes in work flexibility.

## 4. Discussion

As far as we know, this is one of very few studies that examined work flexibility defined as ability to work at home, take time off, and change schedule using GSS-QWL data from all the available years, and the first study to assess the relationship of HRQL and perceived flexibility indicators. Our results demonstrated several statistically significant associations among work flexibility and well-being indicators and can inform the well-being outcomes of the ongoing changes in the workplace, work, and workforce [44]. For example, continued changes in work organization practices and work arrangements are increasingly affecting the workplace, technological advances increasingly affect how work is conducted, and changes in demographics and skills are increasingly impacting the workforce. To understand and address these changes, the NIOSH Future of Work Initiative [45] describes an integrated approach to address worker well-being and prioritize forthcoming future of work research and resulting activities. A recent publication elaborates on the NIOSH perspective on the future of work [46].

We found that the prevalence of all three flexibility indicators we used remained relatively stable over time. In comparison, in the European Union countries, overall work flexibility is increasing [47]. However, the prevalence of work flexibility among countries is not fully comparable because the types of flexible work practices and the extent of their applications vary. Work flexibility is also specific to the culture, economic conditions, and national level work policies [48]. With the exception of a few countries including Sweden, Denmark, and the Netherlands, work practices are more flexible in the United States than in other developed economies in terms of changes in location and schedule [49]. During the past year, the ability to work remotely increased in the United States. For example, in May 2020, 35.4% of workers teleworked [50]. However, it is hard to predict what the prevalence of telework will be in the longer term.

Our descriptive analyses with pooled data showed that 36% of those who responded they were able to work at home reported job stress. This could reflect the blurred lines between work and home, the perception that workers need to work even harder to prove they earned the right to work remotely, or higher demands in the types of jobs that permit working from home. Findings from the United States and Europe have demonstrated that work flexibility can promote work–family balance, and, in turn, job satisfaction, but it can also intensify work–family conflict by blurring the division between work and non-work domains [25,51,52].

We found that across work arrangements, contractors reported the highest percentages for all flexibility indicators. This is the group that includes independent contractors, consultants, and freelance workers and houses the largest share (67%) of workers in non-standard arrangements. There is evidence that contractors also seem to be better off than workers in other arrangements in terms of reported well-being [38]. Technological advances might add more workers to this category and allow higher levels of work flexibility among contractors. Similar trends are observed in Europe [47]. The prevalence of different types of non-standard work arrangements in the United States seems to be increasing [53]. While understanding of the complexities of newer work arrangements, such as those involving electronically mediated platforms, remains challenging, improved definitions of all work arrangements would help. The importance of work arrangements is recognized by the NIOSH Healthy Work Design and Well-being cross-sector program that includes this topic in its three priority areas [54]. In addition, NIOSH economists and epidemiologists are pursuing the collection of more detailed data on work arrangements by adding related questions in national surveys that clarify the specific elements of these arrangements beyond the broad categories that are currently available in GSS-QWL and other national surveys. This detailed information is organized in a taxonomy [55]. The elements of this taxonomy also include aspects of flexibility examined in this study.

Regression analyses on job stress showed that working at home increased the likelihood of reporting job stress but taking time off and changing one’s schedule reduced the odds of job stress. Part of the increase in job stress could be attributed to overwork resulting from taking work home. As we could not account for that in this study, we did not distinguish among those who work at home as part of a contractual agreement versus those who are overworked and have to take work home. The reported odds of job satisfaction increased for all flexibility indicators. These results point to the mixed well-being outcomes associated with work flexibility.

For women in particular, all flexibility indicators were associated with an increased reported likelihood of job stress and days with activity limitations, and a decreased reported likelihood of healthy days. This points to the difference among male and female respondents in terms of healthy days. A recent publication elaborates on these issues [46]. It highlights challenges associated with gender, including that even though women comprised over half of the US workforce in 2018, they continue to face disparities in terms of workplace policies and earnings levels [56]. Working mothers, one of the fastest-growing segments of the US workforce, continue to struggle to balance work and family demands, especially if their earnings are low and their organizational support is low [57,58,59,60]. In terms of overall family interference with work, we found that working at home and changing one’s schedule increased the reported likelihood of job stress and days with activity limitations and decreased the reported likelihood of job satisfaction and healthy days.

We found that with age, the reported likelihood of job stress decreased, and job satisfaction and healthy days increased. Additionally, work flexibility increases with age. This is welcoming news for the aging workforce. In the future, the multigenerational and aging workforce may face disproportionate challenges in terms of physical and cognitive health. For example, the proportion of older workers is expected to continue to grow through 2050 [61]. When older workers suffer injuries and illness, their recovery is often slower [62].

We found that family income was associated with an increased reported likelihood of job stress and job satisfaction, pointing to mixed results, as well as a decreased reported likelihood of healthy days and days with activity limitations. Higher paying jobs may be more complex and have higher demands. Additionally, higher family income might imply the presence of a working spouse and higher needs for flexibility. Health status was associated with a decreased reported likelihood of job stress and days with activity limitations, and an increased reported likelihood of job satisfaction and healthy days.

The major contributions of this study included assessing the trends of three work flexibility indicators over time, the population prevalence rates by flexibility indicators and selected demographic and socioeconomic characteristics, and the well-being outcomes of flexibility. This is the only study that used GSS-QWL data from 2002 to 2018 and well-being indicators that included healthy days and days with activity limitations. We found that perceived work flexibility had not improved significantly during the time period examined. Our findings on the association between work flexibility and well-being are important for understanding how worker well-being may change in the future, as flexible work practices are currently expected to continue to rise. Additionally, work flexibility reflects one aspect of work arrangements, which is one of the core emphasis areas in the NIOSH Future of Work initiative [33]; the types of work arrangements have also continued to increase.

### Limitations

Several limitations of our study relate to limitations of the data we used. Apart from the problems of working with subjective measures of flexibility, the data we used are cross-sectional and include a relatively small number of observations, which limits the ability to control for many covariates. In terms of specific variables, as mentioned in the discussion section, GSS-QWL includes only summary definitions of work arrangements. The GSS-QWL question about working at home does not reflect the variety of other locations a person could work and could have resulted in an underestimate of location flexibility in our results. Finally, we only had information on the respondents’ main job and not all their jobs. In terms of the prevalence of flexibility indicators across the survey years, we understand that the macroeconomic conditions during and after the Great Recession of 2007–2009, especially unemployment, likely affect our results. Similarly, we did not control for workload or demand, which could explain to some extent the effect of work intensification on the relationship between working at home and job stress. However, we did control for the number of hours worked. Another shortcoming is our inability to study other well-being measures like that of subjective well-being [63]. The GSS-QWL data do not include items measuring hedonic well-being, while the item on evaluative well-being was added in 2018. The pooled data prohibit us from making across-years comparisons of the associations of work flexibility and well-being. While controlling for macroeconomic factors is beyond the scope of this study, it is a gap that could be addressed in future analyses.

## 5. Conclusions

This study demonstrated the importance of work flexibility for well-being, using three flexibility indicators (working at home, taking time off, and changing one’s schedule) and four well-being indicators (job stress, job satisfaction, healthy days, and days with activity limitations). Key takeaways include that the ability to change one’s schedule was associated with reduced reported likelihood of job stress and that we found a strong association between being able to take time off and three well-being outcomes, including a reduced reported likelihood of job stress, and an improved reported likelihood of job satisfaction and healthy days.

## Figures and Tables

**Table 1:** Flexibility by demographic and socioeconomic characteristics, pooled GSS-QWL data.

Demographic Characteristics	Flexibility Indicators (% in Columns)			All (% in Column)
	Work at Home	Take Time off	Change Schedule	
Age (significance)	***	***	***	
Mean	46.0	43.5	43.8	42.8
Total	2325	5298	3188	7315
Sex (significance)	*	**	***	
Female	(48.9)	(50.8)	(48.8)	(51.7)
Total	2335	5310	3196	7339
Race and ethnicity (significance)	***	***	***	
Hispanic	(7.4)	(9.7)	(8.4)	(10.6)
Non-Hispanic				
White	(75.9)	(67.5)	(74.0)	(66.0)
Black	(7.5)	(13.3)	(8.6)	(13.1)
Asian or Pacific Islander	(5.3)	(6.4)	(5.8)	(07.0)
Multiracial	(3.7)	(2.6)	(2.8)	(02.7)
American Indian or Alaskan Native	(0.3)	(0.5)	(0.4)	(0.5)
Total	2320	5285	3182	7302
Education (significance)	***	***	***	
1–7 years of school	(0.8)	(1.2)	(1.2)	(1.4)
8 years of school	(0.7)	(0.9)	(0.8)	(1.0)
9–11 years of school	(3.4)	(7.3)	(5.7)	(7.8)
High school graduate	(15.5)	(25.4)	(21.7)	(25.5)
13–15 years of school	(23.0)	(29.7)	(28.9)	(29.9)
Bachelor’s degree	(27.1)	(19.9)	(22.9)	(18.8)
Postgraduate	(29.9)	(15.6)	(18.7)	(15.6)
Total	2333	5305	3192	7329
Family Income (significance)	***	***	***	
USD 10,000 or less	(4.0)	(6.6)	(5.4)	(7.0)
USD 10,001–25,000	(10.0)	(18.5)	(15.0)	(19.0)
USD 25,001–50,000	(25.0)	(31.0)	(29.0)	(32.0)
USD 50,001–75,000	(24.0)	(20.0)	(21.0)	(19.0)
USD 75,000 and above	(37.0)	(25.0)	(30.0)	(23.0)
Total	2153	4886	2911	6757
Health status (significance)	**	***	**	
Overall health good	(87.9)	(87.4)	(88.2)	(86.1)
Total	2321	5303	3191	7319
Marital status (significance)	***	***	***	
Married and living with spouse	(56.6)	(48.4)	(51.4)	(47.3)
Total	2333	5309	3195	7337
Family-work interference (significance)	***	**	***	
Family interferes with work	(66.1)	(43.0)	(60.0)	(28.7)
Total	2312	5306	3182	7312
Hours worked per week (significance)	***	***	-	
Mean Total	43.5 2335	40.4 5310	42.0 3196	42 7315

Note: *, **, and ***, respectively, denote statistical significance at the 10%, 5%, and 1% level.

**Table 2:** Well-being by flexibility indicator, pooled GSS-QWL data.

Well-Being Indicators	Flexibility Indicators (% in Columns)			All (% in Columns)
	Work at Home	Take Time off	Change Schedule	
Job stress (significance)	***	***	-	
Stressed	(36.0)	(25.3)	(30.4)	(31.1)
Total	2318	5300	3188	7311
Job satisfaction (significance)	***	***	***	
Satisfied	(94.4)	(92.3)	(92.3)	(89.8)
Total	2318	5299	3187	7309
Number of days in good physical health (significance)	-	***	-	
Mean	27.3	27.7	27.5	27.4
Total	2314	5292	3184	7297
Number of days in good mental health (significance)	-	***	-	
Mean	26.8	27.0	26.7	26.5
Total	2320	5279	3176	7281
Healthy days (significance)	-	***	-	
Mean	24.4	24.9	24.5	24.2
Total	2314	5279	3176	7281
Number of days with activity limitations (significance)	-	***	-	
Mean	1.4	1.2	1.3	1.4
Total	2318	5293	3184	7299

Note: *** denotes statistical significance at the 1% level.

**Table 3:** Flexibility by NORA industry sector, pooled GSS-QWL data.

NORA Sector	Flexibility % in Rows			All % in Column
	Work at Home	Take Time Off	Change Schedule	
Agriculture, forestry, and fishing	53.2	72.7	68.8	1.0
Construction	27.5	76.3	70.7	7.0
Healthcare and social assistance	19.8	70.4	50.3	14.0
Manufacturing	19.6	74.4	47.6	11.0
Mining	10.5	73.7	63.6	0.3
Oil and gas extraction	11.1	66.7	16.7	0.1
Public safety	11.3	68.8	35.5	2.2
Services	32.6	74.0	57.7	46.4
Transportation, warehousing, and utilities	13.0	67.6	45.0	6.2
Wholesale and retail trade	19.0	71.2	53.1	12.2
All sectors	25.8	72.8	54.3	100
Total (numbers in columns)	7176	7142	5765	7176

**Table 4:** Flexibility by work arrangement, pooled GSS-QWL data.

Work Arrangement	Flexibility % in Rows			All % in Column
	Work at Home	Take Time Off	Change Schedule	
Significance	***	-	***	
Contractor	64.3	76.0	81.2	13.7
On call	21.7	72.3	55.8	2.8
Temporary	0.2	65.8	33.9	1.1
Under contract	27.2	69.1	54.1	3.0
Standard	26.8	72.4	49.4	79.4
Total (numbers in column)	2309	5286	3176	7293

Note: *** denotes statistical significance at the 1% level.

**Table 5:** Ability to work at home and well-being, pooled GSS-QWL data.

	Job Stress Odds Ratio	Job Satisfaction Odds Ratio	Healthy Days IRR	Days with Activity Limitations IRR
Work at home	1.22 *** 1.08–1.37	1.65 *** 1.33–2.04	0.99 ** 0.97–1.00	1.09 0.90–1.30
Age	0.99 *** 0.98–0.99	1.03 *** 1.02–1.03	1.00 *** 1.00–1.00	1.00 0.99–1.00
Sex ^A^	1.38 *** 1.23–1.54	1.15 0.92–1.30	0.95 *** 0.94–0.97	1.12 0.95–1.32
Race and ethnicity ^B^				
Hispanic	0.68 *** 0.56–0.82	1.22 0.93–1.60	1.05 *** 1.03–1.06	0.61 ** 0.43–0.87
Black	0.60 *** 0.50–0.72	0.96 0.75–1.22	1.04 *** 1.02–1.06	1.15 0.86–1.52
Native American	0.77 0.35–1.68	1.44 0.44–5.03	1.00 0.93–1.08	0.77 0.54–1.12
Asian	0.64 ** 0.45–0.91	0.80 0.49–1.32	1.04 * 1.00–1.09	0.77 0.54–1.13
Multiracial	0.99 0.81–1.22	0.79 0.59–1.05	0.97 ** 0.94–0.99	1.12 0.89–1.41
Education	1.05 ** 1.00–1.19	1.05 0.98–1.12	1.00 0.99–1.00	0.91 ** 0.85–0.99
Family income	1.105 0.97–1.12	1.22 *** 1.11–1.35	1.01 *** 1.01–1.02	1.00 0.91–1.10
Health status ^C^	0.64 *** 0.55–0.74	2.19 *** 1.80–2.68	1.16 *** 1.14–1.18	0.50 *** 0.42–0.60
Marital status ^D^	1.05 * 1.01–1.08	0.95 * 0.89–0.99	0.99 ** 0.99–0.99	1.04 0.99–1.08
Family-work interference ^E^	1.38 *** 1.30–1.47	0.83 *** 0.75–0.91	0.98 *** 0.97–0.98	1.09 * 1.02–1.19
Hours worked per week	1.03 *** 1.02–1.03	1.01 *** 1.00–1.02	1.00 * 1.00–1.00	1.11 ** 1.01–1.23
Constant	0.06 *** 0.02–0.13	0.14 *** 0.05–0.41	21.03 *** 19.01–23.25	8.37 *** 2.70– 25.90

Notes: *, **, and ***, respectively, denote statistical significance at the 10%, 5%, and 1% level; ^A^: reference group male; ^B^: reference group non-Hispanic White; ^C^: reference group not good health; ^D^: reference group not married and living with spouse; ^E^: reference group no family-work interference.

**Table 6:** Ability to take time off and well-being, pooled GSS-QWL data.

	Job Stress Odds Ratio	Job Satisfaction Odds Ratio	Healthy Days IRR	Days with Activity Limitations IRR
Take time off	0.44 *** 0.39–0.50	2.15 *** 1.81–2.55	1.02 *** 1.01–1.04	0.76 *** 0.63–0.92
Age	0.99 *** 0.99–0.99	1.03 *** 1.02–1.03	1.00 *** 1.00–1.00	1.00 0.99–1.00
Sex ^A^	1.31 *** 1.17–1.47	1.13 0.95–1.34	0.96 *** 0.94–0.97	1.09 0.92–1.29
Race and ethnicity ^B^				
Hispanic	0.65 *** 0.53–0.78	1.27 * 0.96–1.67	1.05 *** 1.03–1.07	0.61 *** 0.44- 0.89
Black	0.60 *** 0.50–0.72	0.92 0.72–1.18	1.04 *** 1.02 - 1.06	1.16 0.87–1.55
Native American	0.76 0.34–1.68	1.45 0.43–4.92	1.00 0.93–1.08	0.81 0.31–2.13
Asian	0.57 ** 0.42–0.86	0.87 0.53–1.45	1.04 ** 1.00–1.07	0.78 0.54 -1.12
Multiracial	0.95 0.76–1.16	0.81 0.61–1.09	0.97 ** 0.95–0.99	1.13 0.89–1.43
Education	1.09 *** 1.04–1.14	1.08 * 1.01–1.16	0.99 0.99–1.00	0.93 ** 0.86–0.99
Family income	1.08 ** 1.01 –1.16	1.21 *** 1.10–1.33	1.01 ** 1.00–1.02	1.01 0.92–1.11
Health status ^C^	0.64 *** 0.55–0.76	2.09 *** 1.71–2.55	1.17 *** 1.14–1.22	0.53 *** 0.44–0.63
Marital status ^D^	1.03 * 0.99–1.07	0.94 ** 0.89–0.99	0.99 *** 0.99–1.00	1.03 0.97–1.08
Family-work interference ^E^	1.32 *** 1.24–1.41	0.89 * 0.80–0.97	0.98 *** 0.97–0.98	1.11 ** 1.00–1.22
Hours worked per week	1.02 *** 1.02–1.03	1.01 *** 1.01–1.02	1.00 ** 1.00–1.00	0.99 *** 0.99–0.99
Constant	0.07 *** 0.03–0.16	0.69 *** 0.02–0.21	20.91 *** 18.91–23.12	9.61 *** 3.03–30.56

Note: *, **, and ***, respectively, denote statistical significance at the 10%, 5%, and 1% level. ^A^: reference group male; ^B^: reference group non-Hispanic White; ^C^: reference group not good health; ^D^: reference group not married and living with spouse; ^E^: reference group no family-work interference.

**Table 7:** Ability to change one’s schedule and well-being, pooled GSS-QWL data.

	Job Stress Odds Ratio	Job Satisfaction Odds Ratio	Healthy Days IRR	Days with Activity Limitations IRR
Change schedule	0.80 *** 0.71–0.91	1.62 *** 1.33–1.96	1.01 0.99–1.02	0.88 0.73–1.06
Age	0.99 *** 0.99–0.99	1.03 *** 1.02–1.04	1.00 *** 1.00–1.00	1.00 0.99–1.01
Sex ^A^	1.32 *** 1.17–1.50	1.06 0.88–1.28	0.95 *** 0.94– 0.96	1.11 0.91–1.34
Race and ethnicity ^B^				
Hispanic	0.75 *** 0.61–0.93	1.10 0.81–1.49	1.04 ** 1.01–1.06	0.72 0.47–1.10
Black	0.63 *** 0.51–0.77	0.84 0.64 -1.09	1.03 *** 1.01–1.06	1.20 0.86–1.66
Native American	0.84 0.35–1.99	1.83 0.42–8.01	1.03 0.95–1.11	0.80 0.20–3.15
Asian	0.78 0.53–1.16	0.85 0.48–1.53	1.04* 1.00– 1.08	0.88 0.56–1.37
Multiracial	0.98 0.79–1.26	0.67 ** 0.49–0.93	0.96 * 0.93–0.99	1.12 0.83–1.50
Education	1.09 *** 1.04–1.15	1.06 0.98–1.14	1.00 0.99–1.01	0.92 ** 0.85–1.00
Family income	1.04 0.96–1.22	1.25 *** 1.12–1.38	1.01 *** 1.01–1.02	1.01 0.91–1.13
Health status ^C^	0.58 *** 0.49–0.70	2.14 *** 1.70–2.68	1.18 *** 1.14–1.22	0.52 *** 0.42–0.64
Marital status ^D^	1.02 0.98–1.06	0.96 0.90–1.02	0.99 ** 0.99–0.99	1.05 0.98–1.11
Family-work interference ^E^	1.41 *** 1.31–1.51	0.80 *** 0.73–0.89	0.97 *** 0.97–0.98	1.13 ** 1.02–1.26
Hours worked per week	1.03 *** 1.02–1.03	1.01 *** 1.01–1.02	1.00 0.99–1.00	0.99 *** 0.99–1.00
Constant	0.05 *** 0.02–0.14	0.10 *** 0.03–0.30	21.67 *** 19.82–23.70	6.03 *** 1.65–22.03

Note: *, **, and ***, respectively, denote statistical significance at the 10%, 5%, and 1% level. ^A^: reference group male; ^B^: reference group non-Hispanic White; ^C^: reference group not good health; ^D^: reference group not married and living with spouse; ^E^: reference group no family-work interference.

## Data Availability

The data used in this paper are publicly available and can be downloaded free of cost from the University of Chicago NORC website http://gss.norc.org/ (accessed on 20 December 2020).
